# Force Depression in Plantar Flexors Exists Equally in Plantar Flexed and Dorsiflexed Regions

**DOI:** 10.3389/fphys.2017.00183

**Published:** 2017-03-24

**Authors:** Atsuki Fukutani, Jun Misaki, Tadao Isaka

**Affiliations:** ^1^Faculty of Kinesiology, University of CalgaryCalgary, AB, Canada; ^2^Japan Society for the Promotion of Science, Postdoctoral Fellowships for Research AbroadKusatsu, Japan; ^3^Research Organization of Science and Technology, Ritsumeikan UniversityKusatsu, Japan; ^4^Graduate School of Sport and Health Science, Ritsumeikan UniversityKusatsu, Japan; ^5^Faculty of Sport and Health Science, Ritsumeikan UniversityKusatsu, Japan

**Keywords:** fascicle length, pennation angle, joint angle, ultrasonography, electrical stimulation

## Abstract

Isometric muscle force attained during isometric contractions decreases after active shortening compared to that attained during purely isometric contractions. This phenomenon is called residual force depression. The aim of this study was to examine whether residual force depression occurs in human plantar flexors in both plantar flexed and dorsiflexed region. In addition, the magnitude of fascicle shortening was evaluated because not only muscle force but also fascicle shortening during active shortening are considered to affect force depression. Eleven male subjects were recruited. All muscle contractions were evoked by muscle belly-electrical stimulation. In the reference trials, isometric plantar flexion (PF) was performed at 0° and 15° of PF. In the residual force depression trials, the following two contractions were conducted: (1) muscles were activated isometrically at 15° of dorsiflexion, then actively shortened to 0° of PF (long condition) and (2) muscles were activated isometrically at 0° of PF, then actively shortened to 15° of PF (short condition). Isometric joint torque obtained 4.9 s after the onset of contraction was compared between the reference and residual force depression trials at the same joint angle to calculate the magnitude of residual force depression. At the same time point, fascicle length and pennation angle were obtained from ultrasonographic images to examine whether the muscle architecture affected residual force depression. As a result, residual force depression was confirmed in both the long and short length conditions (long: 87.1 ± 9.1%, short: 92.1 ± 7.8%) while the magnitude was not different (*p* = 0.182). The fascicle length and pennation angle were not different between the reference and residual force depression trials (*p* = 0.291–0.906). These results indicate that residual force depression occurs in the physiological range of motion in the human plantar flexors, and this phenomenon is not related to muscle architecture. In addition, joint angle dependence of the residual force depression was not observed between long and short muscle length conditions.

## Introduction

The isometric force attained at a given muscle length and activation level is smaller after active shortening compared to the isometric force attained at a pure isometric contraction (Abbott and Aubert, 1952). This phenomenon is called force depression/residual force depression (Edman et al., 1993; Herzog, 2004). This phenomenon is widely observed in isolated whole muscle (Abbott and Aubert, 1952; Herzog and Leonard, 1997), single fiber (Sugi and Tsuchiya, 1988; Granzier and Pollack, 1989), and myofibril (Joumaa and Herzog, 2010; Trecarten et al., 2015) preparations. Although the precise mechanism(s) is debatable, the most acceptable mechanism at present is active shortening-induced inhibition of cross-bridge formation (Maréchal and Plaghki, 1979; Herzog, 2004). Specifically, because actin filaments have small but some compliance (Huxley et al., 1994; Higuchi et al., 1995), and consequently, the actin filaments are pulled during active shortening due to muscle force, the shape of actin filaments is deformed. This leads to dislocation of the binding site of the myosin head, which causes inhibition of cross-bridge formation. Inhibition of cross-bridge formation will lead to a reduction in the total number of cross-bridges. As a result, the isometric force after active shortening decreases. This concept supported by previous studies that showed that the magnitude of residual force depression was strongly related to the magnitude of force produced during active shortening (Herzog and Leonard, 1997; De Ruiter et al., 1998). This concept is reasonable because larger force produces a larger deformation of the actin filament, which should cause a stronger inhibition. As the force produced during an active shortening increases, the deformation of actin filaments becomes larger, which causes larger inhibition of cross-bridge formation. Consequently, a larger magnitude of residual force depression is observed.

Because the phenomenon of residual force depression has been observed in various muscle preparations consistently, it is reasonable to assume that residual force depression also occurs in everyday human movements and affects its performance. Several studies have examined whether residual force depression occurs in human movements, and confirmed substantial residual force depression in the adductor pollicis (De Ruiter et al., 1998; Lee and Herzog, 2003; Rousanoglou et al., 2007), quadriceps femoris (Lee et al., 1999), and tibialis anterior (Tilp et al., 2011). Therefore, it is obvious that residual force depression occurs in the physiological range of human movements. Moreover, in the study by Tilp et al. (2011), the effect of muscle architecture (i.e., fascicle length and pennation angle) on residual force depression was examined, and these parameters were not found to affect residual force depression. However, because muscle architectural characteristics differ among muscles (Wickiewicz et al., 1983; Lieber and Fridén, 2000), the effect of muscle architectural parameters on residual force depression should be examined in each muscle to obtain better insights on residual force depression in human movements.

Therefore, the aim of this study was to examine whether residual force depression occurs in human plantar flexors in relation to the characteristics of muscle architecture. In addition, the magnitude of residual force depression was compared between different muscle length conditions. It was hypothesized that residual force depression is not related to the muscle architecture in plantar flexors in line with the previous study that used the tibialis anterior (Tilp et al., 2011). In addition, because joint torque (muscle force) is larger in long muscle conditions than in short muscle conditions within the physiological range of motion due to the force-length relationship of the plantar flexors (Kawakami et al., 1998; Maganaris, 2001), the magnitude of residual force depression would be larger in the longer muscle condition because the magnitude of residual force depression was shown to be force-dependent (Herzog and Leonard, 1997; De Ruiter et al., 1998).

## Materials and methods

### Subjects

Eleven healthy young men (mean ± standard deviation: age, 24.8 ± 3.1 years; height, 1.72 ± 0.04 m; body mass, 65.7 ± 6.3 kg) voluntarily participated in the present study. The purpose and associated risks of the study were explained to each volunteer, and written informed consent was obtained from all the participants. The Ethics Committee on Human Research of Ritsumeikan University approved this study (IRB-2016-007), and the study was conducted according to the principles stated in the Declaration of Helsinki.

### Experimental setup

Ankle plantar flexors of the right leg were adopted as the target muscles in this study. Subjects lay supine on a dynamometer (Biodex; SAKAImed, Tokyo, Japan). The ankle, knee, and hip joint angles were set at 0° (i.e., anatomical position were defined as 0°). The upper body and upper thigh were fixed on the dynamometer to restrict redundant movements. Throughout the experiment, the knee and hip joint angles remained the same. The ankle joint was fixed on the attachment of the dynamometer using a non-elastic band. The ankle joint angle was moved using the dynamometer. The center of rotations of the ankle joint and the attachment of dynamometer were carefully aligned visually. The following two conditions were tested: the first was the short muscle condition (short condition), in which isometric joint torque was recorded at 15° of plantar flexion (PF), while the second was the long muscle condition (long condition), in which isometric joint torque was recorded at 0° of PF. In each condition, a reference trial (pure isometric contraction) and residual force depression trial (i.e., isometric contraction after active shortening) were conducted to calculate the magnitude of residual force depression by comparing the isometric joint torque obtained at the same joint angle and activation level as that in the above two trials. In this experiment, all muscle contractions were evoked using electrical stimulation (SEN-3401; Nihon Kohden, Tokyo, Japan) to induce stable joint torque responses. Stimulation electrodes (4 × 5 cm) were placed on the muscle bellies on the upper side of both gastrocnemii and the lower side of the soleus to equally activate the major plantar flexors. The stimulation parameters were as follows: pulse frequency, 50 Hz; pulse duration, 0.5 ms; and train duration, 5 s. To determine the intensity of electrical stimulation, maximal voluntary isometric contraction in the plantar flexors was conducted with the ankle joint angle at 0°. The highest isometric joint torque recorded during this contraction was set at 100% intensity. The intensity of electrical stimulation was adjusted to evoke 25% of the maximal intensity at the corresponding joint angle. This electrical stimulation intensity was applied to all the contractions.

**Figure 1:**
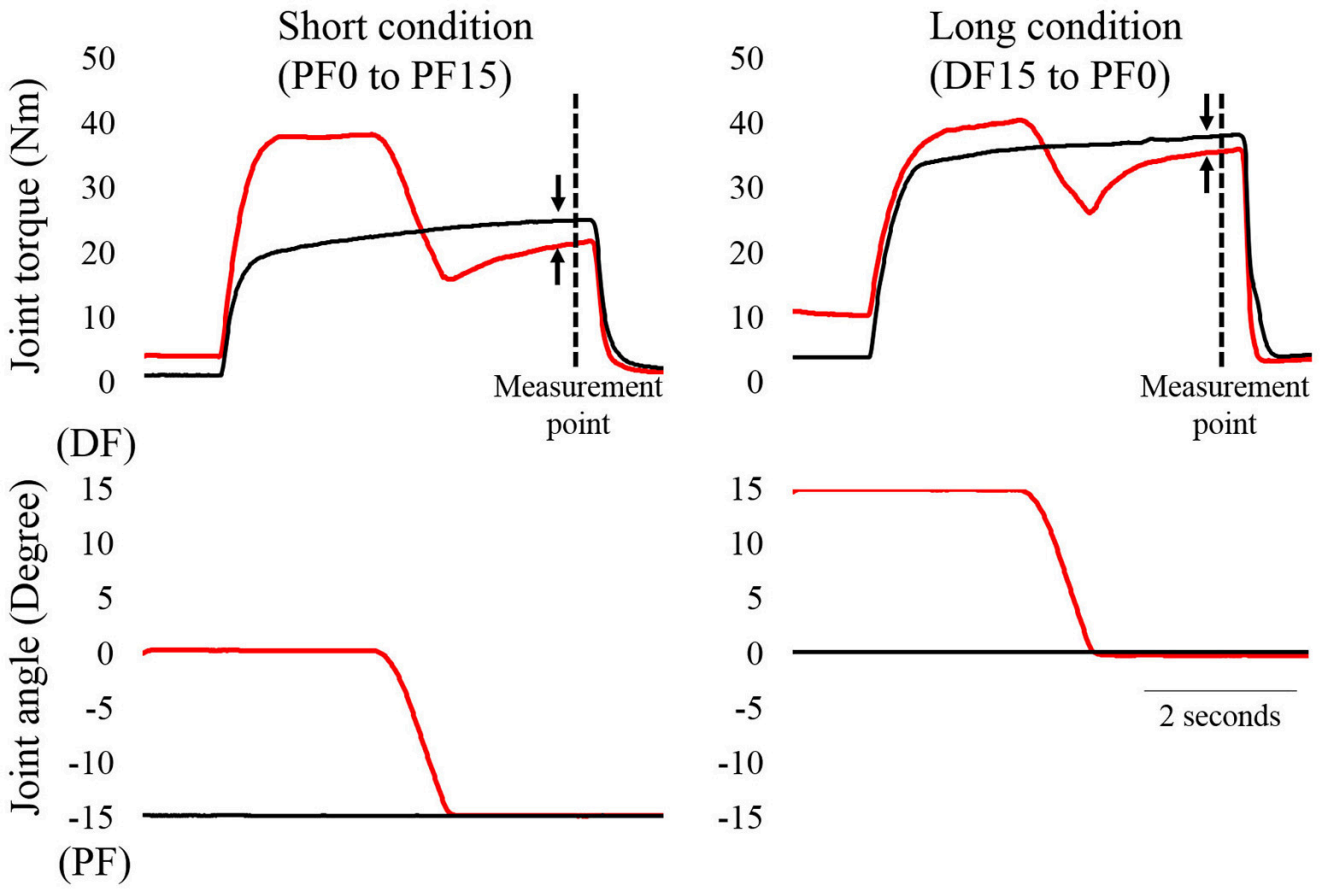
**Time course changes in joint torque and joint angles as a function of time (*N* = 1)**. The left panel shows the short condition and the right panel shows the long condition. The black line shows the reference trial and the red line shows the residual force depression trial. The magnitude of residual force depression was calculated 4.9 s after the onset of contractions (dotted line). The arrows indicate the magnitude of residual force depression.

A typical example of the experimental trials is shown in Figure 1. In the short condition, isometric joint torque was compared at 15° of PF (i.e., 4.9 s after the onset of contraction). In the reference trial, the ankle joint angle was set at 15° of PF. Electrical stimulation was then applied for 5 s. In the residual force depression trial, the ankle joint angle was set at 0° of PF. Electrical stimulation was then applied for 5 s. Two seconds after the onset of contraction, the ankle joint angle was rotated to 0° of PF at a joint angular velocity of 20°/s. In the long condition, isometric joint torque was compared at 0° of PF. In the reference trial, the ankle joint angle was set at 0° of PF. Electrical stimulation was then applied for 5 s. In the residual force depression trial, the ankle joint angle was set at 15° of dorsiflexion (DF). Electrical stimulation was then applied for 5 s. Two seconds after the onset of contraction, the ankle joint angle was rotated to 0° of PF with a joint angular velocity of 20°/s. The control trials were conducted first, and the residual force depression trials were conducted second. The sequence of the short and long conditions was randomized. The interval between trials was at least 2 min to avoid the effects of muscle fatigue on the next trial. After the end of all trials, maximal voluntary isometric contraction in the plantar flexors at 0° was conducted again to confirm whether muscle fatigue occurred or not, and no decrease in joint torque was observed (pre experiments: 140.2 ± 21.4 Nm, post experiments: 141.8 ± 22.8 Nm). During these trials, subjects were instructed to maintain a relaxed state to avoid redundant voluntary contractions.

### Joint torque and ultrasonographic measurements

**Figure 2:**
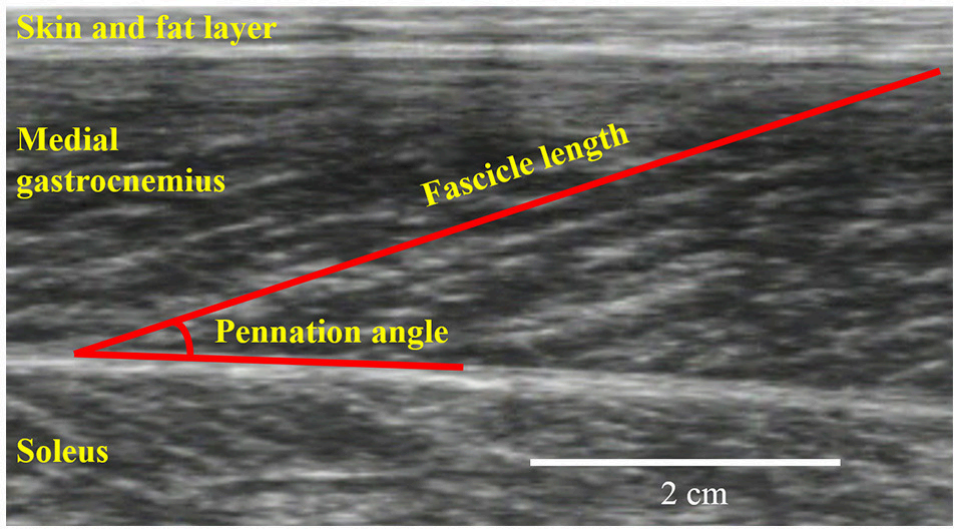
**An example for ultrasonographic measurements**. Fascicle length and pennation angle were obtained from the medial gastrocnemius.

The joint torque and joint angle were recorded at a sampling frequency of 4,000 Hz (Power lab 16/30; ADInstruments, Bella Vista, Australia). Ultrasonographic measurements (SSD-3500; Aloka, Tokyo, Japan) were performed at the same time as joint torque measurements. The fascicle length and pennation angle of the medial gastrocnemius were measured using a linear array probe (UST-5710; Aloka, Tokyo, Japan) with a sampling frequency of 30 Hz. The fascicle length was defined as the straight distance between the intersection composed of the superficial aponeurosis and fascicle and the intersection composed of the deep aponeurosis and fascicle (Figure 2). The pennation angle was defined as the internal angle formed by the fascicle and deep aponeurosis. The acquired images were analyzed using Image J 1.47v software (National Institutes of Health, Bethesda, MD, USA).

Isometric joint torques recorded 4.9 s after the onset of contraction were used to calculate the magnitude of residual force depression in both the short and long conditions as absolute (Nm) and relative (%) measurements. Fascicle lengths and pennation angles recorded 4.9 s after the onset of contraction were obtained to compare the muscle architecture between the reference and residual force depression trials. In addition, the magnitude of angular impulse (joint torque [Nm] × time [s]) produced during active shortening phase was calculated as the index of the magnitude of muscle force produced during the active shortening. Moreover, the magnitude of fascicle shortening during the active shortening phase was also calculated.

### Statistics

The magnitude of residual force depression as absolute and a relative measurements was compared between the short and long conditions using paired *t*-tests. Fascicle lengths and pennation angles obtained during the reference and residual force depression trials were compared by paired *t*-tests to confirm whether muscle architectural properties affected the differences in joint torque. Moreover, the angular impulse and the magnitude of fascicle shortening during the active shortening phase were compared between the short and long conditions by paired *t*-test. Statistical analyses were performed using SPSS version 20 software (IBM, Tokyo, Japan) with the level of statistical significance set at *p* < 0.05. All values are shown as mean ± standard deviation.

## Results

### Residual force depression

**Figure 3:**
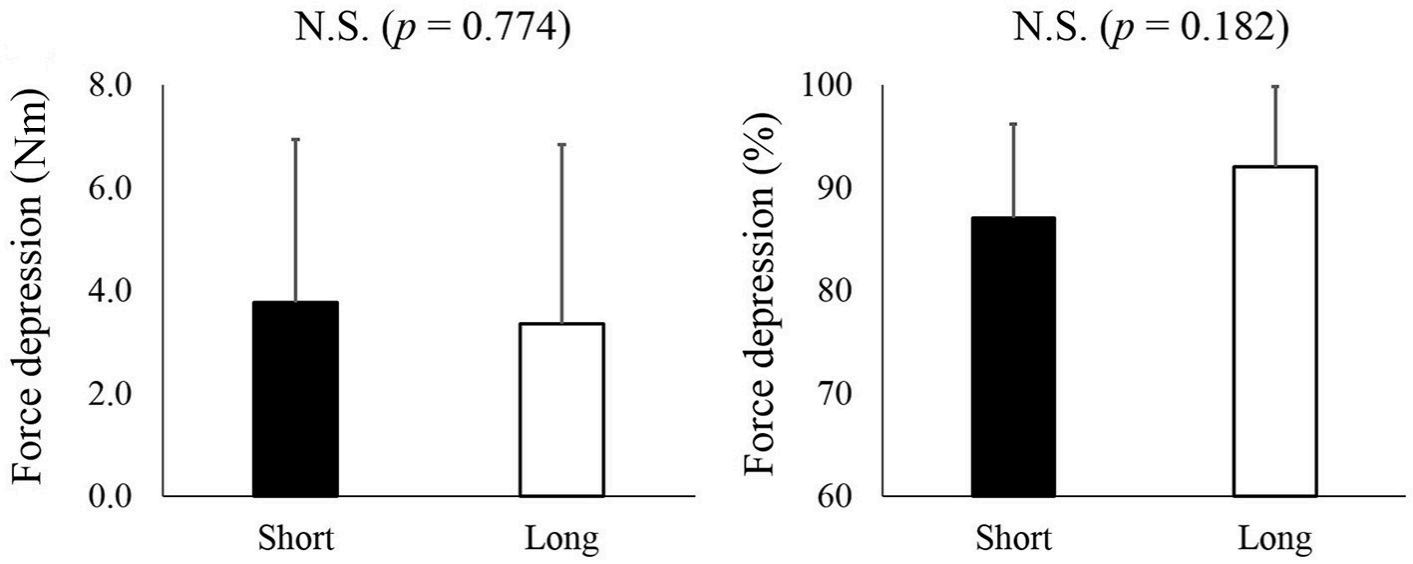
**The magnitude of residual force depression**. The left panel shows the magnitude of residual force depression calculated as an absolute value while the right panel shows the magnitude of residual force depression calculated as a relative value. The black bars represent the short condition while white bars represent the long condition.

Residual force depression calculated in an absolute manner did not differ between the short (3.8 ± 3.2 Nm) and long conditions (3.4 ± 3.2 Nm) (*p* = 0.774) (Figure 3, left panel). Similarly, the magnitude of residual force depression calculated in a relative manner did not differ between the short (87.1 ± 9.1%) and long conditions (92.1 ± 7.8%) (*p* = 0.182) (Figure 3, right panel).

### Muscle architecture

**Figure 4:**
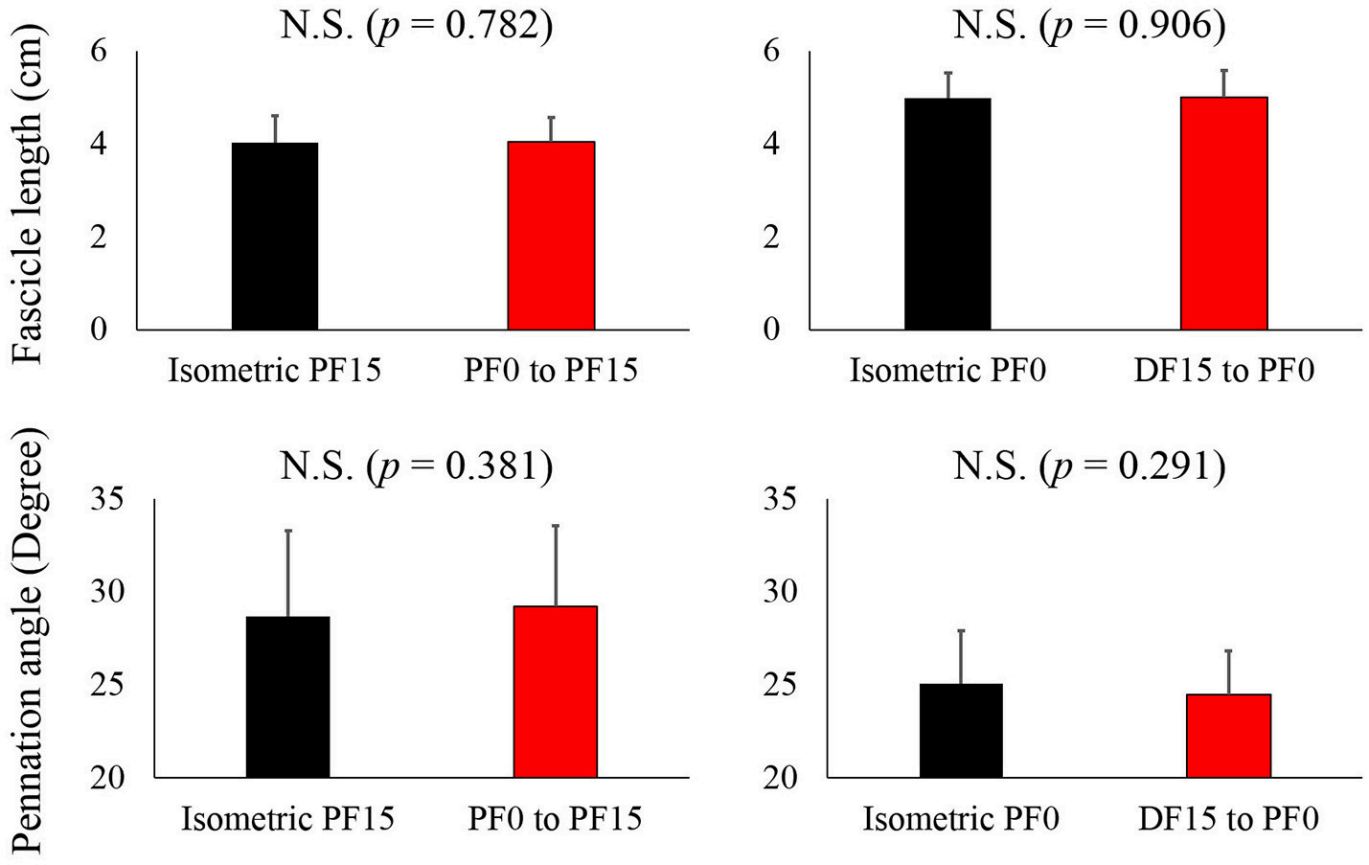
**Comparison of the fascicle length and pennation angle between the reference and residual force depression trials**. The black bars represent reference trials while red bars represent the residual force depression trials.

For fascicle length, the paired *t*-test revealed no significant difference between reference and residual force depression trials in both the short and long conditions (*p* = 0.782 for the short condition and *p* = 0.906 for the long condition) (Figure 4, upper panel). Similarly, the pennation angle was not different between reference and residual force depression trials in both the short and long conditions (*p* = 0.381 for the short condition and *p* = 0.291 for the long condition) (Figure 4, lower panel).

### Angular impulse and fascicle shortening during the active shortening phase

**Figure 5:**
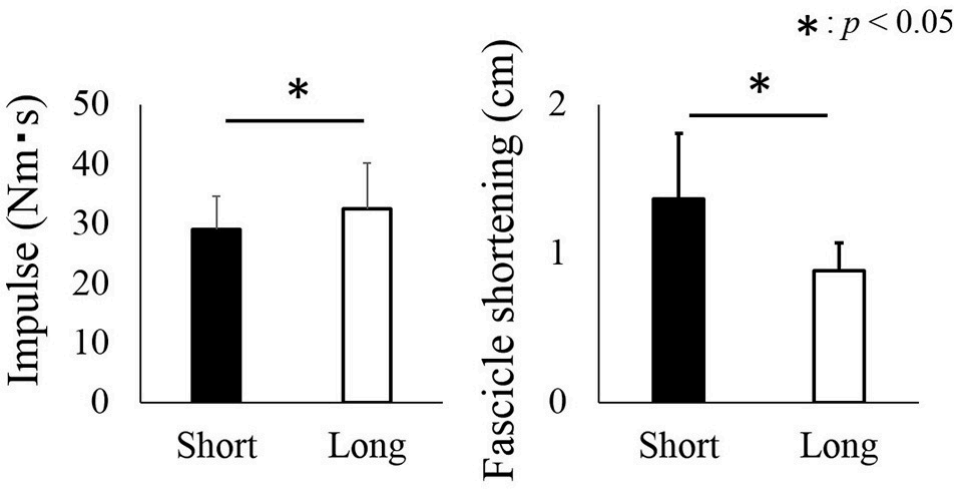
**Impulse and fascicle shortening attained during the active shortening phase**. The black bars represent the short condition while white bars represent the long condition. The asterisk (^*^) indicates a significant difference (*p* < 0.05) between the short and long conditions.

The angular impulse was significantly larger in the long condition (32.5 ± 7.6 Nm·s) than in the short condition (29.0 ± 5.6 Nm·s) (*p* = 0.016) (Figure 5, left panel). On the other hand, the magnitude of fascicle shortening was larger in the short condition (1.4 ± 0.4 cm) than in the long condition (0.9 ± 0.2) (*p* = 0.004) (Figure 5, right panel).

## Discussion

The purpose of this study was to examine whether residual force depression occurs in human plantar flexors and whether the magnitude of residual force depression differs between short and long muscle length conditions in relation to the characteristics of muscle architecture. We found a substantial decrease in joint torque in human plantar flexors in both conditions, but no significant differences in the magnitude of residual force depression were found between the two conditions, which seems to contradict our working hypothesis. This observation is further discussed below.

### Effect of the force produced and fascicle shortening

Although the magnitude of angular impulse as an index of muscle force produced during the active shortening phase was larger in the long condition than in the short condition, the magnitude of residual force depression did not differ between the two conditions. This result seems to contradict that of previous studies (Herzog and Leonard, 1997; De Ruiter et al., 1998). A possible reason for the above contradiction could be related to the muscle architectural properties of human plantar flexors. In this study, the range of motion for the long and short conditions was identical (15° of DF to 0° of PF for the long condition and 0° of PF to 15° of PF for the short condition). However, the magnitude of fascicle shortening would not necessarily be identical between the short and long conditions. In fact, the observed fascicle shortening during active shortening was significantly larger in the short condition than in the long condition. This larger fascicle shortening at the same magnitude of joint angle change could be caused by the difference in moment arm of the plantar flexors. Previous studies reported that the magnitude of moment arm was larger in the PF region than in the DF region (Rugg et al., 1990; Maganaris et al., 1998). Geometrically, larger moment arm induces larger shortening at a given angle change. Based on these geometrical properties of human plantar flexors, larger fascicle shortening should be observed in the short condition. Because it is well known that not only muscle force but also the magnitude of muscle (fascicle) shortening during the active shortening phase affects the magnitude of residual force depression (Herzog and Leonard, 1997), the observed larger shortening could have masked the effect of a larger force in the long condition on the magnitude of residual force depression.

### Effect of the muscle architecture

In this study, a substantial decrease in joint torque was observed in the residual force depression trials compared to the reference trials, although the muscle architectural parameters did not differ between both conditions. Therefore, this decrease in joint torque may be caused by the mechanism(s) of residual force depression, possibly inhibition of the cross-bridge formation due to the deformation of actin filaments (Maréchal and Plaghki, 1979; Herzog, 2004). This result is in line with that of a previous study on the tibialis anterior (Tilp et al., 2011). However, this result seems unusual based on the concept that the tendon (series elastic component with respect to muscle) is elongated by muscle force and the magnitude of elongation of a tendon increases as muscle force (joint torque) increases (Fukashiro et al., 1995). The fascicle should be longer in the residual force depression trials due to smaller tendon elongation, which was related to the small muscle force. This would be caused by a small difference in the joint torque (3.8 ± 3.2 Nm for the short condition and 3.4 ± 3.5 Nm for the long condition). These values correspond to less than 4% of the maximal voluntary isometric joint torque at 0° of PF and would not be sufficient to induce substantial tendon length changes. Similar to our study and that by Tilp et al. (2011), other studies examining residual force enhancement in human muscles reported that muscle architectural properties did not change significantly although joint torque was different between reference and residual force enhancement trials (Seiberl et al., 2010; Tilp et al., 2011; Power et al., 2013). Based on these similar findings, our results indicating that joint torque was different while fascicle length and pennation angle were similar between the two conditions seem reasonable and feasible.

### Other possible factors related to residual force depression

Previously, not only the deformation of actin filaments, but also sarcomere length non-uniformity (Edman et al., 1993) and active shortening-induced metabolic byproducts (Granzier and Pollack, 1989) have been discussed as the reason for residual force depression. Regarding sarcoma length non-uniformity, residual force depression was confirmed even in the single sarcomere preparation (Trecarten et al., 2015). Because the influence of sarcomere length non-uniformity is absent in the single sarcomere preparation, sarcoma length non-uniformity should not be the primary factor for residual force depression. Regarding active shortening-induced metabolic byproducts, it is known that the influence of residual force depression quickly disappears once active force reaches to zero (Abbott and Aubert, 1952) although metabolic byproducts would not disappear within such a short duration. Thus, this factor would be discarded. In addition, recently, titin-based residual force depression mechanism has been proposed (Rode et al., 2009). If this concept is correct, residual force depression occurs only when titin passive force exists. However, in this experiment, we confirmed substantial residual force depression even in the ascending limb where the influence of passive force derived from the titin should be small or negligible. Our finding is in line with previous studies (Herzog and Leonard, 1997; De Ruiter et al., 1998). Therefore, this mechanism is unlikely the primary factor for residual force depression.

### Limitation

Even in the reference trial of this study, (i.e., purely isometric contraction judging from joint angle changes), muscle fascicle shortened during the early phase of contractions due to possibly tendon elongation or eliminating slack (Fukashiro et al., 1995; Herbert et al., 2015; Hirata et al., 2016). This fascicle shortening may have induced residual force depression. However, considering the fact that residual force depression is dependent on force applied to the actin filament (Maréchal and Plaghki, 1979; Herzog, 2004) and that force during the early phase of contraction is relatively low, the influence of residual force depression induced by this initial phase of isometric contraction (judging from joint angle changes) is small. In addition, because this fascicle shortening occurred in both the reference and residual force depression trials in this study, it is unlikely that this fascicle shortening substantially affected our main result.

## Conclusions

We confirmed that substantial residual force depression occurred in human plantar flexors within the physiological range of motion. Thus, this muscle property should be taken into consideration when analyzing human movements. In addition, we found that the magnitude of residual force depression was similar between long and short muscle conditions. This may be caused by larger fascicle shortening in short muscle conditions than in long muscle conditions. This muscle architectural property-related modulation of residual force depression implies that experiments using human muscles are required to obtain a better understanding of the significance of residual force depression in human movements.

## Ethics statement

This study was carried out in accordance with the recommendations of the Committee on Human Research of Ritsumeikan University with written informed consent from all subjects. All subjects gave written informed consent in accordance with the Declaration of Helsinki. The protocol was approved by the Committee on Human Research of Ritsumeikan University.

## Author contributions

AF and JM performed the experiments and analyzed data. AF, JM, and TI wrote the paper.

## Funding

This study was partially supported by the Grant-in-Aid for Challenging Exploratory Research (16K13009) and Postdoctoral Fellowship for Research Abroad (183).

### Conflict of interest statement

The authors declare that the research was conducted in the absence of any commercial or financial relationships that could be construed as a potential conflict of interest.
